# Plastome analysis unveils Inverted Repeat (IR) expansion and positive selection in Sea Lavenders (*Limonium*, Plumbaginaceae, Limonioideae, Limonieae)

**DOI:** 10.3897/phytokeys.175.61054

**Published:** 2021-04-01

**Authors:** Ashwini M. Darshetkar, Satish Maurya, Changyoung Lee, Badamtsetseg Bazarragchaa, Gantuya Batdelger, Agiimaa Janchiv, Eun Ju Jeong, Sangho Choi, Ritesh Kumar Choudhary, Soo-Yong Kim

**Affiliations:** 1 Biodiversity & Palaeobiology Group, Agharkar Research Institute, Pune 411 004, India; 2 International Biological Material Research Center, Korea Research Institute of Bioscience and Biotechnology, Daejeon, 34141, South Korea; 3 Department of Environment & Forest Resources, Chungnam National University, Daejeon 34134, South Korea; 4 Botanic Garden and Research Institute, Mongolian Academy of Sciences, Ulaanbaatar 13330, Mongolia; 5 Department of Biology, Ulaanbaatar State University, Ulaanbaatar 13343, Mongolia; 6 Department of Plants & Biomaterials Science, Gyeongsang National University, Jinju 52725, South Korea; 7 S.P. Pune University, Pune 411 007, India

**Keywords:** Intron loss, IR expansion, positive selection, pseudogenisation, *ycf1*

## Abstract

The genus *Limonium*, commonly known as Sea Lavenders, is one of the most species-rich genera of the family Plumbaginaceae. In this study, two new plastomes for the genus *Limonium*, viz. *L. tetragonum* and *L. bicolor*, were sequenced and compared to available *Limonium* plastomes, viz. *L. aureum* and *L. tenellum*, to understand the gene content and structural variations within the family. The loss of the *rpl16* intron and pseudogenisation of *rpl23* was observed. This study reports, for the first time, expansion of the IRs to include the *ycf1* gene in *Limonium* plastomes, incongruent with previous studies. Two positively selected genes, viz. *ndhF* and *ycf2*, were identified. Furthermore, putative barcodes are proposed for the genus, based on the nucleotide diversity of four *Limonium* plastomes.

## Introduction

The family Plumbaginaceae of the order Caryophyllales is highly diverse, rich in species and displays a cosmopolitan distribution with its maximum diversity in the temperate areas of the northern hemisphere (Kubitzki 1993). It is sister to Polygonaceae (Lledó et al. 1998; Chase et al. 2016) and further classified into two subfamilies: Limonioideae (formerly Staticoideae) and Plumbaginoideae (Lledó et al. 1998, 2001; Hernández-Ledesma et al. 2015). Limonioideae is further divided into two tribes, Limonieae (consisting of 24 genera) and the monotypic Aegialitideae, whereas Plumbaginoideae consists of four genera. Limonioideae is a sub-cosmopolitan group distributed mostly in the Mediterranean and Indo-Turanian regions, but a few genera have also diversified in the Southern Hemisphere. *Limonium* Mill., *Acantholimon* Boiss. and *Armeria* (DC.) Willd., all belonging to Limonioideae, are the most species-rich genera, comprising 80–90% of the species in Plumbaginaceae (Koutroumpa et al. 2018).

The genus *Limonium* Mill., popularly known as sea lavenders, belongs to the subfamily Limonioideae and tribe Limonieae (Kubitzki 1993; Lledó et al. 2005; Malekmohammadi et al. 2017). The genus is represented by ca. 600 species and is the sole genus of Plumbaginaceae exhibiting a sub-cosmopolitan distribution (Koutroumpa et al. 2018). The genus comprises several ornamental and medicinally-important species (Malekmohammadi et al. 2017). *Limonium* are herbs or shrubs growing in saline or metal-rich soils, mostly in coastal areas (Kubitzki 1993). Variation of reproductive systems (sexual and apomixis), as well as events like hybridisation and polyploidy, complicate the delimitation of most of the species of *Limonium* (Kubitzki 1993; Cowan et al. 1998; Palacios et al. 2000; Akhani et al. 2013; Róis et al. 2016).

Certain phylogenetic studies tried to resolve the relationships within *Limonium* at a global scale (Lledó et al. 2005); however, many others were confined to either a specific geographic area or some specific sections of *Limonium* (Palacios et al. 2000; Lledó et al. 2005; Lledó et al. 2011; Akhani et al. 2013; Róis et al. 2016). More recently, Malekmohammadi et al. (2017) tried to resolve the phylogenetic relationships within *Limonium* including 76 species of the Mediterranean region. They found two well-supported clades for subgenera *Limonium* and *Pteroclados* (Boiss.) Pignatti, which confirmed the earlier findings by Lledó et al. (2005, 2011). Their study was based on one nuclear and several plastid loci, but lacked comprehensive sampling. Koutroumpa et al. (2018) carried out a phylogenetic study of Plumbaginaceae which included 23 genera of the family with an emphasis on the genus *Limonium* (201 spp.), based on three chloroplast and one nuclear marker. The study again confirmed most of the previous molecular phylogenies and led to the proposal of new sections and altering some of the existing sections. However, taxonomic difficulties, diversity in reproductive modes and sub-cosmopolitan distribution of *Limonium* necessitate further studies on the genus.

With the advent of sequencing technologies, the availability of large genome-scale data has made it easier to understand phylogeny and detect polyploidy events (Gompert and Mock 2017; Thomas et al. 2017; McKain et al. 2018). Five plastomes have been sequenced for Plumbaginaceae thus far, viz. *Limonium sinense* (Girard) Kuntze (Li et al. 2020), *L. aureum* (L.) Chaz. (Zhang et al. 2020), *L. tenellum* (Turcz.) Kuntze (Yao et al. 2019) and one each of *Ceratostigma* Bunge and *Plumbago* L. The recent phylogenomic study to understand the evolution of Caryophyllales incorporated plastome sequences of *L. tenellum*, *Plumbago auriculata* Lam. and *Ceratostigma willmottianum* Stapf (Yao et al. 2019). The study reported that all Plumbaginaceae members, except *Limonium*, exhibit expanded IR to accommodate *ycf1*. However, no studies have been carried out to understand and compare the structure, composition and evolution of the plastome within Plumbaginaceae and *Limonium* in particular.

The present study reports the plastome sequences of two Asian *Limonium* species, viz. *L. tetragonum* (Thunb.) Bullock and *L. bicolor* (Bunge) Kuntze and compares the structure, composition and diversity within the genus by combining them with other available plastomes. *L. tetragonum* is a biennial species characterised by a spicate inflorescence, yellow corolla, acute calyx with pink at the base, white in upper parts and distributed in Japan, Korea, New Caledonia and Primorye (Ohwi 1965; POWO 2021). *Limonium bicolor* is a perennial species, characterised by a paniculate inflorescence, yellow corolla, somewhat rounded calyx, pink to purplish at base, white in upper parts and distributed in China and Mongolia (Wu and Raven 1996; POWO 2021). The former is known for its anti-cancerous properties (Kong et al. 2008; Bae et al. 2016), while the latter is most studied for its salt glands (Li et al. 2019; Lu et al. 2020). An attempt has also been made to unravel the structural variations within Plumbaginaceae plastomes and propose molecular markers for easier discrimination of *Limonium* species.

## Material and methods

### Sampling and sequencing

Leaf samples of *Limonium bicolor* and *L. tetragonum* were collected from Meneng steppe of Dornod Province of Mongolia (Voucher No. KRIB 0070251) in June 2015 and from the coastal area of Ulsan City of the Republic of Korea (Voucher No. KRIB 0086343) in April 2018, respectively. The samples were deposited at the Herbarium of Korea Research Institute of Bioscience and Biotechnology (KRIB). DNA extraction was carried out from dried leaves using the DNeasy Plant Mini Kit (QIAGEN, Cat. No. 69104) according to the manufacturer’s protocol. For both the plastomes, a 550 bp DNA TruSeq Illumina (Illumina, San Diego, CA, USA) sequencing library was constructed. After the library preparation, the DNA samples were run in a single lane of an Illumina HiSeq 10X with a read length of 151 bp.

### Assembly and annotation

The raw reads obtained after Illumina sequencing were analysed using FastQC V0.11.7 (Andrews 2010) software to ensure the quality of the reads and Phred score. The assembly was carried out using NOVOPlasty V4.2 (Dierckxsens et al. 2017). Forward and reverse reads with a read length of 150 bp and insert size of 300 bp, as well as seed sequence (*rbcL* of *L. tetragonum*), were used as input. *De novo*, as well as reference-based assembly, were carried out. The plastome sequence of *L. aureum* (MN623109) was used as a reference for the assembly of both the plastomes. The assembly and orientation of the Inverted Repeats (IRs), Large Single Copy (LSC) and Small Single Copy (SSC) regions were confirmed by NCBI blast and graphic view using Geneious prime 2020.2.2 (https://www.geneious.com). The assembled plastomes were annotated using Geneious prime 2020.2.2 (https://www.geneious.com) and Geseq – Annotation of Organellar Genomes (Tillich et al. 2017). The transfer RNAs (tRNAs) were identified with tRNA-scan-SE (Lowe and Chan 2016). Graphical maps of both plastomes were visualised using OGDRAW (Lohse et al. 2013).

### Comparative plastome analysis

All Plumbaginaceae plastomes available so far were included for comparison. Four plastomes of *Limonium*, viz. *L. aureum* (MN623109), *L. tenellum* (MK397871), *L. tetragonum*, *L. bicolor* and *Ceratostigma willmottianum* (MK397862), as well as *Plumbago auriculata* (MH286308), were included in the analysis. The plastome of *L. sinense* was not included in any of the analyses due to ambiguities observed in the assembly. The selected six plastomes were aligned using a Geneious prime 2020.2.2 plugin MAFFT v.7.450 (Katoh and Standley 2013). The contraction and expansion of IRs at the junction sites were examined and plotted using IRscope (Amiryousefi et al. 2018). Percentage sequence identity for all six plastomes was plotted using MultiPIPMaker (http://pipmaker.bx.psu.edu/pipmaker/) considering *L. tetragonum* as a reference (Schwartz et al. 2003).

### Identification of Simple Sequence Repeats (SSRs) and repeats

Simple Sequence Repeats across the four *Limonium* plastomes were detected using MISA online server (Beier et al. 2017). The minimum repeat units for mono-, di-, tri-, tetra-, penta- and hexanucleotide repeats were set as 10, 5, 4, 3, 3 and 3, respectively, whereas forward, reverse, palindromic and complementary repeats were identified using the programme REPuter (Kurtz et al. 2001). The threshold for repeat length was set to 30 with more than 90% similarity and the hamming distance was set to 3.

### Nucleotide diversity

The four *Limonium* plastomes were aligned using Geneious prime 2020.2.2 plugin MAFFT v.7.450 (Katoh and Standley 2013). The aligned sequences were curated manually and, due to the presence of ambiguous *ycf1* region in *L. tenellum* at IRb/ SSC junction, the sequences were trimmed for all four taxa before the analysis. Nucleotide diversity and sliding window analysis were performed using the software DnaSP v.6.12.01 (Rozas et al. 2017).

### Codon usage

The percentage codon usage of protein-coding regions of all four *Limonium* plastomes was calculated using Geneious prime 2020.2.2. To examine the frequency and uniformity of Synonymous codon and codon biases, the Relative Synonymous Codon Usage (RSCU) was also determined in DnaSP v.6.12.01 software (Rozas et al. 2017).

### Positive selection analysis

In order to detect protein-coding genes under selection in the genus *Limonium*, sequences of each gene were aligned using the MAFFT v.7.450 plugin of Geneious prime 2020.2.2. The aligned sequences were again manually checked for an end-to-end alignment. The phylogenetic tree for each protein-coding gene was constructed using the FastTree plugin (Price et al. 2010) of Geneious prime 2020.2.2. The site-specific model was performed using CODEML algorithms (Yang 1998) implemented in EasyCodeML software (Gao et al. 2019). Seven codon substitution models (M0, M1a, M2a, M3, M7, M8 and M8a) were investigated and compared to identify positively selected sites, based on likelihood ratio tests.

### Phylogenomic analysis

A total of 39 plastome sequences, including six from Plumbaginaceae were considered as ingroups for phylogenomic analysis. The outgroup was composed of members of Amaranthaceae. All the plastome sequences were aligned using the MAFFT v.7.450 plugin of Geneious prime 2020.2.2. Maximum Likelihood analysis, based on the best fit model GTR+F+R5, was performed using IQtree 1.6.12-MacOSX (Nguyen et al. 2015). The best likelihood score for the consensus tree was -979751.011.

## Results

### General features of the plastomes

**Figure 1.**
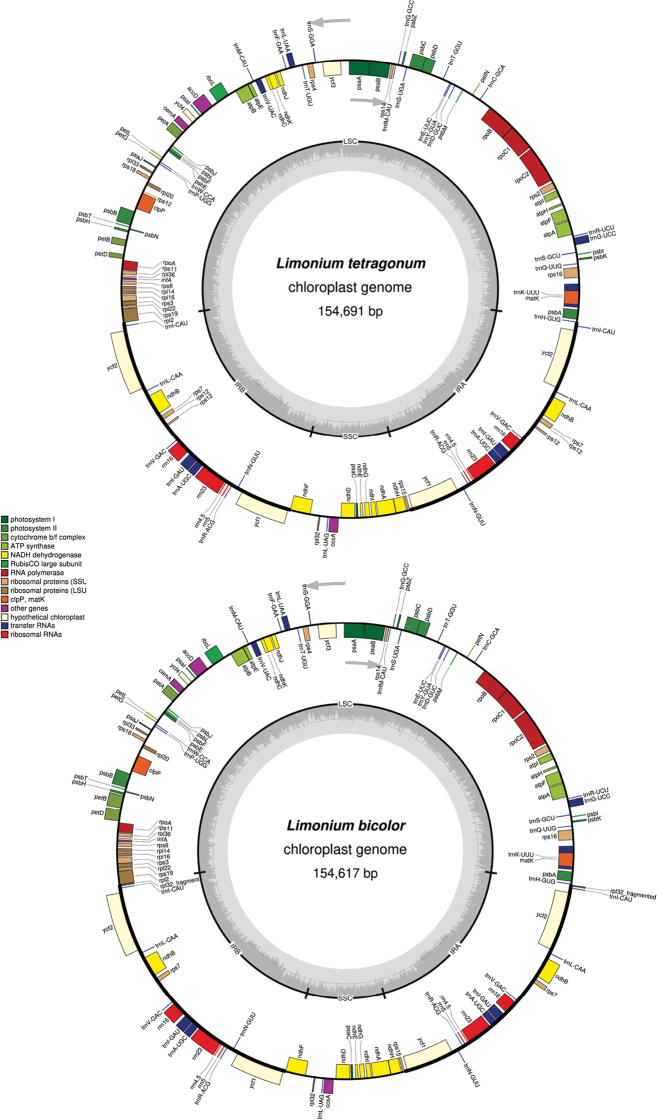
Circular gene map of plastomes of *L. tetragonum* and *L. bicolor*. Genes drawn inside the circle are transcribed clockwise and those outside are counter-clockwise. Genes belonging to different functional groups are shown in different colours. The innermost circle denotes GC content across the plastome.

The average organelle coverage for the plastomes of *L. tetragonum* and *L. bicolor* was 1014X and 1009X, respectively. Plastomes of *L. tetragonum* and *L. bicolor* exhibited a typical quadripartite structure (Fig. 1). Total plastome lengths of *L. tetragonum* and *L. bicolor* were 154,691 bp and 154,617 bp with LSC (84,568 bp and 84,541 bp), SSC (12,997 bp and 12,964 bp) and a pair of IRs (28,563 bp and 28,556 bp), respectively (Table 1). The GC content of both plastomes was 37%. Both the plastomes exhibited 83 protein coding genes, 37 tRNA genes and four rRNA genes (duplicated in IR region) (Tables 1, 2). The assembled and annotated plastomes of *L. tetragonum* and *L. bicolor* were deposited to the NCBI database with accession numbers MW085088 and MW085089, respectively.

**Table 1.** Comparison of plastome features of Plumbaginaceae members.

Species	*Limonium tetragonum*	*Limonium bicolor*	*Limonium aureum*	*Limonium tenellum*	*Ceratostigma willmottianum*	*Plumbago auriculata*
Accession No.	MW085088	MW085089	MN623109	MK397871	NC041261	NC041245
Genome size (bp)	154691	154617	154661	150515	164999	168765
LSC length (bp)	84568	84541	84546	84634	89454	91912
SSC length (bp)	12997	12964	12980	23755	13491	13331
IR length (bp)	28563	28556	28568	21063	31027	31761
No. of genes duplicated in IR	15	15	16	10	15	19
No. of genes	128	128	130	124	127	132
No. of protein coding genes	83	83	83	82	82	84
No. of tRNA genes	37	37	37	36	37	37
No. of rRNA genes	8	8	8	6*	8	8
Total GC content (%)	37	37	37.1	37.1	37.5	37.2

*indicates annotations available as per NCBI database.

**Table 2.** List of genes in the newly-sequenced plastomes of *L. tetragonum* and *L. bicolor*.

Category	Group	Name
Photosynthesis-related genes	Rubisco	*rbcL*
	Photosystem 1	*psaA*, *psaB*, *psaC*, *psaI*, *psaJ*
	Photosystem 2	*psbA*, *psbB*, *psbC*, *psbD*, *psbE*, *psbF*, *psbH*, *psbI*, *psbJ*, *psbK*, *psbL*, *psbM*, *psbN*, *psbT*, *psbZ*
	APT synthase	*atpA*, *atpB*, *atpE*, *atpF*†, *atpH*, *atpI*
	Cytochrome b/f complex	*petA*, *petB*, *petD*, *petG*, *petL*, *petN*
	NADPH Dehydrogenase	*ndhA*†, *ndhB*†*, *ndhC*, *ndhD*, *ndhE*, *ndhF*, *ndhG*, *ndhH*, *ndhI*, *ndhJ*, *ndhK*
Transcription and translation-related genes	Transcription	*rpoA*, *rpoB*, *rpoC1*†, *rpoC2*
	Ribosomal proteins	*rps2*, *prs3*, *rps4*, rps7*, *rps8*, *rps11*, *rps12*†*, *rps14*, *rps15*, *rps16*†, *rps18*, *rps19*
		*rpl2*†, *rpl14*, *rpl16*, *rpl20*, *rpl22*, rpl23^#^*, *rpl33*, *rpl36*
	Translation initiation factor	*infA*
RNA genes	Ribosomal RNA	rrn5*, rrn4.5*, rrn16*, rrn23*
	Transfer RNA	*trnA*-*UGC*†*, *trnC*-*GCA*, *trnD-GUC*, *trnE-UUC*, *trnF-GAA*, *trnfM-CAU*, *trnG-UCC*, *trnH-GUG*, *trnI-GAU*†*, *trnK-UUU*†, trnL-CAA*, *trnL-UAA*, *trnL-UAG*†, *trnM-CAU*, trnN-GUU*, *trnP-UGG*, *trnQ-UUG*, trnR-ACG*, *trnR-UCU*, *trnS-GCU*, *trnS-GGA*, *trnS-UGA*, *trnT-GGU*, *trnT-UGU*, trnV-GAC*, *trnW-CCA*, *trnY-GUA*
Other genes	RNA processing	*matK*
	Carbon metabolism	*cemA*
	Fatty acid synthesis	*accD*
	Proteolysis	*clpP*†
Genes of unknown function	Conserved reading frame	*ycf1*†*, ycf2*, *ycf3*†, *ycf4*

*duplicated genes, †genes with introns, #pseudogenised genes.

### IR expansion and contraction

The IR regions of plastomes are divided by four junctions viz., IRb/LSC, IRb/SSC, IRa/SSC and IRa/LSC. All six plastomes of Plumbaginaceae (including four *Limonium*) were compared for their IR boundaries. The annotations available on NCBI database were used for *Limonium aureum*, *L. tenellum*, *Ceratostigma willmottianum* and *Plumbago auriculata*.

**Figure 2.**
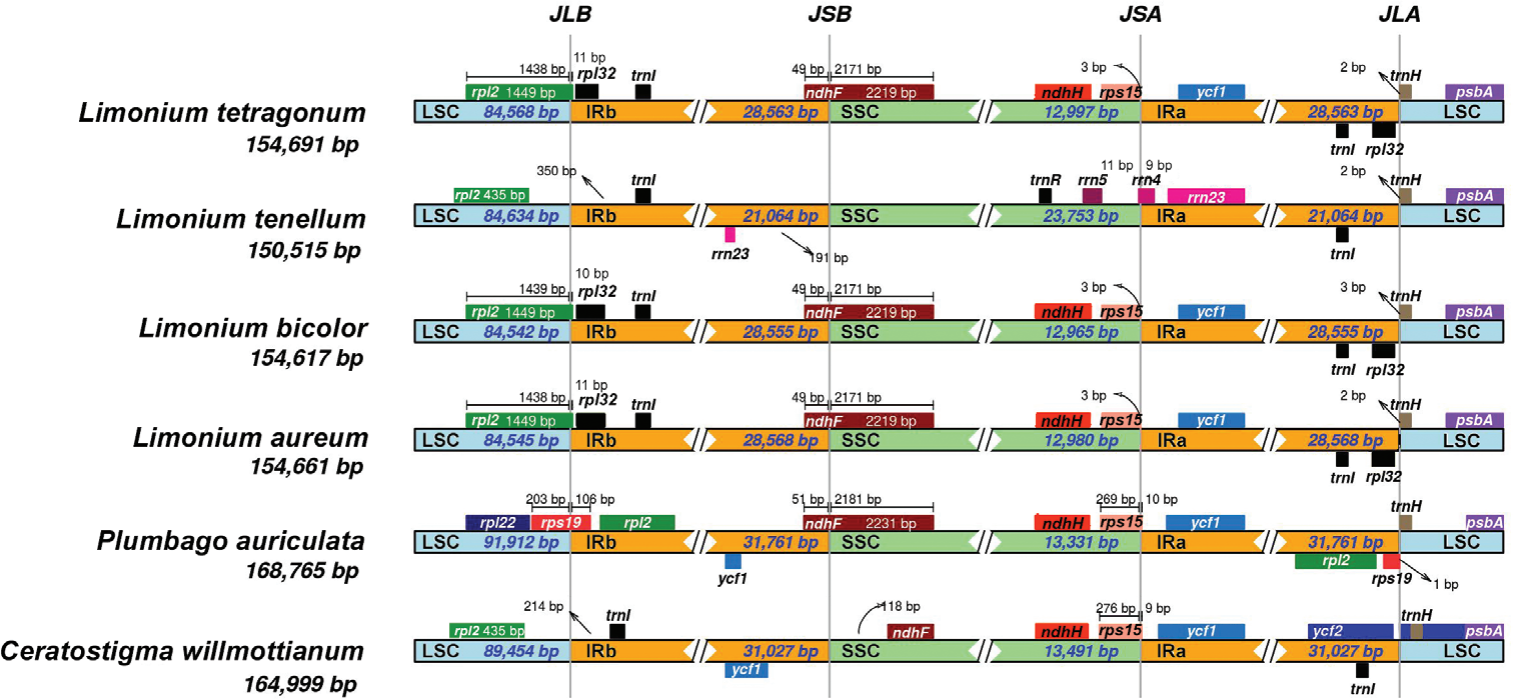
Comparison of IRs of Plumbaginaceae plastomes.

The IRb/LSC junction of three *Limonium* species viz. *L. tetragonum*, *L. bicolor* and *L. aureum* was characterised by the presence of the *rpl2* gene (Fig. 2). The gene also extends in IRb with 11 bp, 10 bp and 11 bp, respectively. However, in *L. tenellum* and *C. willmottianum*, it was exclusively present in the LSC region, 937 bp and 1,041 bp away from the junction, respectively. In *Plumbago*, IR was expanded to include the *rps19* gene at the junction, while *rpl2* was duplicated in the IR region.

The next junction, i.e. IRb/SSC, was characteried by the presence of the gene in *L. tetragonum*, *L. bicolor*, *L. aureum* and *P. auriculata* (49 bp of all *Limonium* species and 51 bp of *Plumbago* in IR region). In *L. tenellum*, the junction exhibited *rrn23* (IR) and *trnR-ACG* (SSC), which could probably be due to wrong assembly or annotation. In *Ceratostigma*, *ndhF* appeared to be shifted to SSC, 118 bp away from IRb border.

IRa/SSC junction of all compared species was characterised by the presence of *rps15* and *ycf1* genes, except in *L. tenellum*. The IRa/LSC junction was characterised by *rpl32* and *trnH* in *L. tetragonum*, *L. bicolor* and *L. aureum*, while in *L. tenellum* and *C. willmottianum*, it was characterised by *ycf2* and *trnH*. In *P. auriculata*, the junction was bordered by *rps19* and *trnH* (Fig. 2).

Plastomes of *L. aureum*, *L. bicolor* and *L. tetragonum* exhibited two copies of *ycf1*, except for *L. tenellum* which exhibited a single copy. All three plastomes are characterised by the *ycf1* gene having a length of 5,298 bp and IR has been expanded to accommodate the *ycf1* gene. *Plumbago* and *Ceratostigma* also exhibited the *ycf1* gene duplicated in IRs. However, the annotation provided for *L. tenellum* (MK397871) exhibits a single copy of *ycf1*.

### Structural comparison

**Figure 3.**
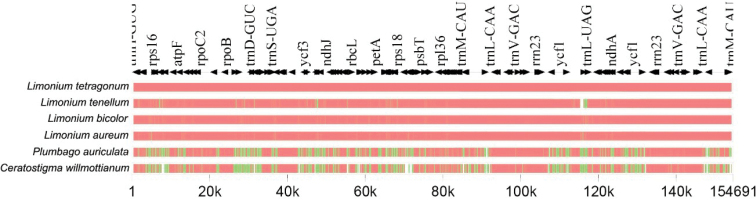
Plastome alignment of Plumbaginaceae members.

Plastomes of *Limonium tetragonum*, *L. tenellum*, *L. bicolor* and *L. aureum* were compared with two Plumbaginaceae plastomes, keeping *L. tetragonum* as a reference. Sequence divergence amongst the four compared *Limonium* plastomes was similar as compared to *Plumbago* and *Ceratostigma*. *Limonium tenellum* exhibited partial deletion at the IRa/LSC junction in the *ycf1* gene (Fig. 3). Our results suggest that the gene *rpl23* has been pseudogenised in all the studied plastomes of the three genera of Plumbaginaceae. The length of *rpl23* in all four *Limonium* plastomes was observed to be 171 bp, 270 bp in *Plumbago* and 50 bp in *Ceratostigma*. A similar loss of intron was observed in *L. aureum*, *L. bicolor* and *L. tetragonum* in our study. In all four compared species of *Limonium*, the length of *rpl16* was 408 bp without any intron. However, the two other genera of Plumbaginaceae, i.e. *Plumbago* and *Ceratostigma*, exhibited the presence of intron. The length of the gene was 1,449 bp in the former, while in the latter, it was 1,469 bp.

### Nucleotide diversity

**Figure 4.**
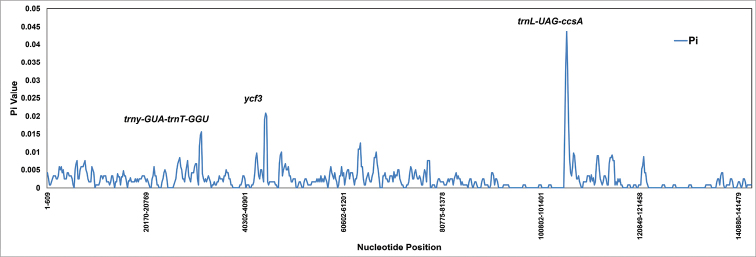
Nucleotide diversity and hotspot regions between *Limonium* plastomes. The X-axis represents the nucleotide position and Y-axis represents nucleotide diversity (Pi).

The nucleotide diversity (Pi) of four *Limonium* plastomes was analysed, except for the *ycf1* region, which was removed due to ambiguous alignment. Sliding window analysis yielded some regions with higher Pi values. High nucleotide diversity was found in two spacer regions viz. *trnY-GUA*-*trnT-GGU*, *trnL-UAG*-*ccsA* and one gene *ycf3* with Pi values 0.015, 0.043 and 0.02, respectively (Fig. 4).

### SSR and repeat analyses

**Figure 5.**
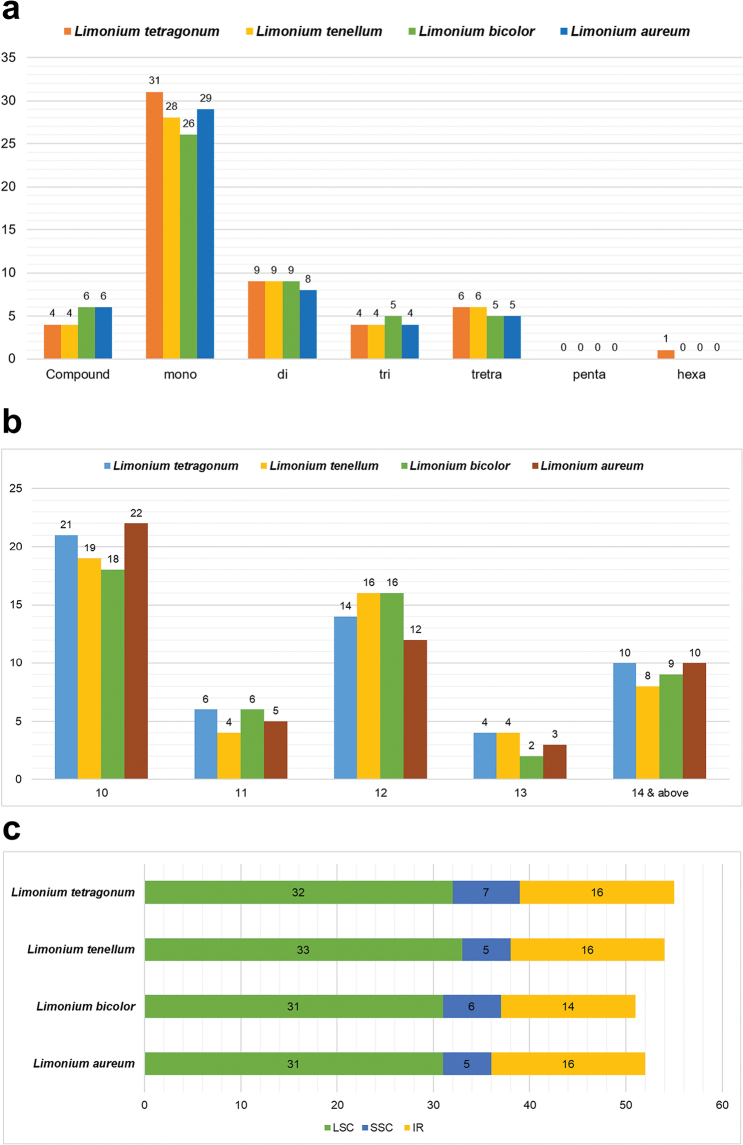
Repeat analysis of four *Limonium* plastomes. a. Types of repeats, b. Size of repeats, c. Position of repeats.

The plastomes of *L. bicolor* exhibited 6 compound, 26 mono-, 9 di-, 5 tri- and 5 tetranucleotide repeats, while *L. tetragonum* exhibited 4 compound, 31 mono-, 9 di-, 4 tri-, 6 tetra- and 1 hexanucleotide repeats (Fig. 5a). The size of repeats ranged from 10 to 168 in *L. bicolor* and 10 to 109 in *L. tetragonum* (Fig. 5b). Repeats in both the plastomes were found to be AT-rich. The highest number of SSRs was found in the LSC region, followed by IR and SSC regions in both the plastomes (Fig. 5c).

All four species of *Limonium* exhibited only forward and palindrome type in REPuter analysis (Suppl. material 1).

### Codon usage

**Figure 6.**
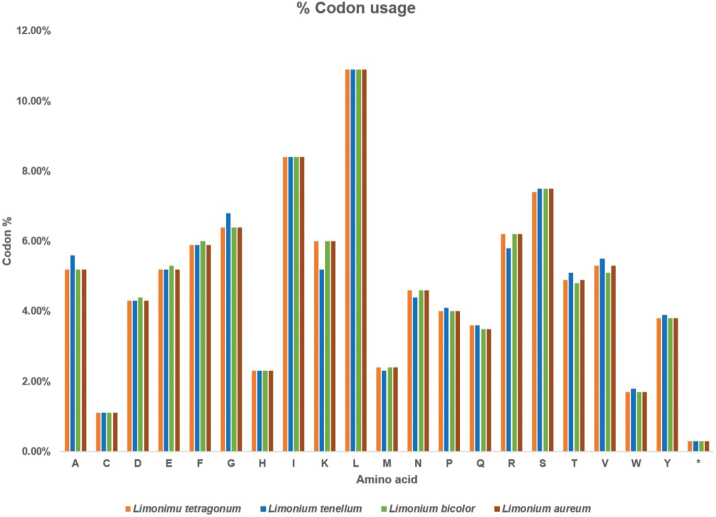
Codon usage of four *Limonium* species. X-axis: Amino acid, Y-axis: codon usage in percentage. * indicates the stop codon.

The four *Limonium* plastomes were compared for their codon usage. The plastomes of *L. tetragonum*, *L. tenellum*, *L. bicolor* and *L. aureum* exhibited 27,290, 24,093, 26,682 and 27,308 codons, respectively. Leucine was the most abundant while Cysteine was the least abundant amino acid in all the compared plastomes (Fig. 6). Codon usage was biased towards A and T in all the compared plastomes. The highest codon preference was 1.88, 1.89, 1.82 and 1.91 while the lowest was 0.33, 0.36, 0.37 and 0.36, respectively, in *L. tetragonum*, *L. tenellum*, *L. bicolor* and *L. aureum* (Suppl. material 2). Codon usage was biased towards A and T in all the compared plastomes.

### Positive selection analysis

A total of 79 consensus protein-coding genes of four *Limonium* species were evaluated with respect to selective pressure. Two genes were found to have undergone positive selection viz. *ndhF* and *ycf2* with ω values 2.2278 and 19.657, respectively (Suppl. material 3).

### Phylogenomic analysis

**Figure 7.**
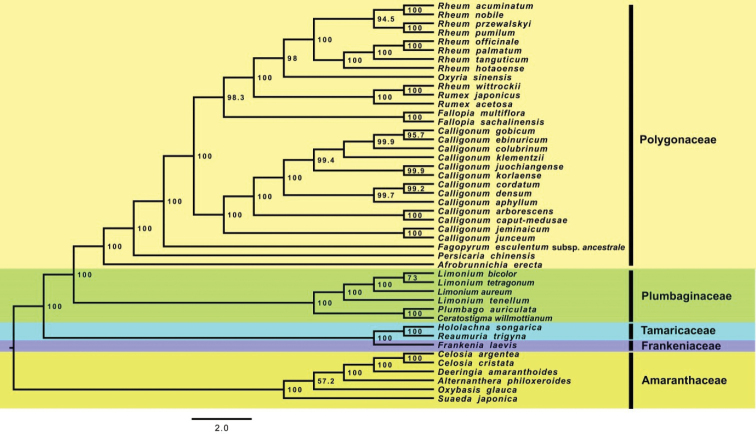
Phylogenomic tree from the Maximum Likelihood analysis showing the placement of the studied four *Limonium* species. Nodal support is represented by bootstrap percentages.

In the phylogenomic analysis, the representatives of *Limonium* formed a strongly-supported monophyletic group (BS = 100), in which *L. bicolor* was recovered as sister to *L. tetragonum* (BS = 73), with *L. aureum* and *L. tenellum* being successive sisters (BS = 100) to the clade of *L. bicolor* and *L. tetragonum*. *Plumbago auriculata* and *Ceratostigma willmottianum* also formed a monophyletic group (BS = 100), sister to the *Limonium* clade (BS = 100). All these made Plumbaginaceae a strongly-supported (BS = 100) monophyletic group (Fig. 7).

## Discussion

In this study, two *Limonium* plastomes were assembled and the structure and composition of four *Limonium* plastomes were compared. The plastomes were conserved in terms of size and structure ranging from 154,617 to 154,691 bp, except for *L. tenellum* with 150,515 bp. Expansion and contraction of IRs/SSC account for huge variation, evolutionary events and also affect the plastome sizes (Zhu et al. 2016; Darshetkar et al. 2019; Maurya et al. 2020). Yao et al. (2019) reported the expansion of IRs in Polygonaceae and Plumbaginaceae members to accommodate *ycf1*, except for the genus *Limonium*. However, the study included a single plastome sequence of *Limonium tenellum*. In all the *Limonium* plastomes, except *L. tenellum*, IR has been expanded to accommodate *ycf1*, unlike that reported by Yao et al. (2019). We assume the single copy of *ycf1* in *L. tenellum* could be a result of erroneous assembly and annotation. Sequencing more plastomes for the genus will help to better understand the arrangement and position of *ycf1* in *Limonium*.

Ribosomal Protein L23 is a protein component of the 60s large ribosomal subunit. The comprehensive study of plastomes of Caryophyllales (Yao et al. 2019) reported that the gene has undergone pseudogenisation at least 11 times in the order. Pseudogenisation has also been reported in the family Polygonaceae, a sister family of Plumbaginaceae (Logacheva et al. 2008). The results of our study corroborate these earlier studies. Transfer of the functional copy of the gene to the nucleus has been reported for many plastid genes in angiosperms (Millen et al. 2001; Jansen et al. 2011; Daniell et al. 2016). The functional copy of *rpl23* might have been transferred to the nucleus, even in the members of Plumbaginaceae as predicted by Yao et al. (2019). Ribosomal Protein L16 codes for a protein component of the 50s ribosomal subunit. The loss of *rpl16* intron was reported in *Limonium gmelinii* Kuntze and *Limonium latifolium* Kuntze (Campagna and Downie 1998). Recently, Yao et al. (2019) reported the loss of *rpl16* intron in *L. tenellum* plastome. Hence, our study confirms the loss of *rpl16* intron on the branch leading to *Limonium* as reported by Yao et al. (2019).

The value of the ratio of synonymous and nonsynonymous substitutions (Ka/Ks or ω) above 1 indicates that the corresponding genes experience positive selection, however, ω values ranging from 0.5 to 1 indicate relaxed selection (Tomoko 1995). The *ndhF* gene was found to be positively selected in the genus *Rheum* L. of Polygonaceae. The study reported that the higher expression levels of *ndhF* were observed in *Rheum* under environmental stress conditions (Li et al. 2016). Most of the *Limonium* species also grow in saline soils, which could be the reason behind the adaptive evolution of the gene. Positive selection of the *ycf2* gene has already been reported in several angiosperms (Jiang et al. 2018; Zhong et al. 2019).

The sampling for the phylogenomic analysis followed the studies of Walker et al. (2018) and Yao et al. (2019). The ingroup consisted of plastomes belonging to the FTPP clade, i.e. Frankeniaceae, Tamaricaceae, Plumbaginaceae and Polygonaceae (they were all recovered as strongly-supported monophyletic groups), while the outgroup consisted of species belonging to Amaranthaceae. The position of the four *Limonium* species studied fits with the earlier studies (Yao et al. 2019). In the present study, phylogenetic position of four Limonium species belonging to subgenus Limonium (Koutroumpa et al. 2018) was studied. A study based on *rbcL*, *trnL* intron and *trnL*-*F* intergenic spacer included two species viz. *L. tenellum* and *L. tetragonum*, which were resolved in the same clade, but were placed into two different subsections, according to Boissier (1848), *Rhodanthae* and *Chrysanthae*, respectively. However, the study treated them under subgenus Limonium (Lledo et al. 2005). Later, Malekmohammadi et al. (2017) studied the phylogeny for the genus, based on several plastid and single nuclear (ITS) markers. The study included *L. tetragonum* and *L. aureum* and both the species were placed in the “*L. aureum* clade”. Recently, Koutroumpa et al. (2018) carried out phylogenetic studies of the genus, based on three chloroplast (*trnL-F*, *matK* and *rbcL*) and one nuclear marker (ITS). Three species, *L. tetragonum*, *L. aureum* and *L. tenellum* were included in the study. These three species were all resolved in the same clade. These species were earlier placed under sect. Plathymenium, *L. tetragonum* and *L. aureum* belonging to subsect. Chrysanthae and *L. tenellum* under subsect. Rhodanthae (Boissier 1848). Earlier studies reported plastome data dealing with single *Limonium* species and placed them as sister to *Plumbago* species (Yao et al. 2019; Li et al. 2020; Zhang et al. 2020). The present phylogenetic study, for the first time, included *L. bicolor* and analysed four *Limonium* plastomes together. However, inclusion of more *Limonium* plastomes would help in understanding the intrageneric relationships.

## Conclusions

The present study has made an effort to understand the structural changes in the plastomes of Plumbaginaceae by including two newly-generated *Limonium* plastome sequences. The study also confirms the loss of *rpl16* intron in the genus *Limonium* and pseudogenisation of the *rpl23* gene in Plumbaginaceae. Our results also revealed, for the first time, the expansion of the IRs to accommodate the *ycf1* gene in *Limonium* as in other Plumbaginaceae members. The annotation available for *L. tenellum* exhibits *ycf1* in the SSC region. Hence, the sequencing of more plastomes would aid in identifying the exact position of *ycf1*. Two positively-selected genes were identified, viz. *ndhF* and *ycf2*. The positive selection of these genes could be linked to the evolution of *ndhF* to adapt to extreme environmental conditions, such as salt stress. It would be interesting to identify the adaptive sites in the *ndhF* amino acid by adding more *ndhF* sequences of *Limonium* species, while the expansion of IRs and accommodation of *ycf2* genes could be related to the re-arrangement of the plastome. The function of *ycf2* is still not clear, but it would be interesting to study the *ycf2* evolution and re-arrangement in the whole order. High nucleotide diversity was observed in two spacer regions *trnY-GUA*--*trnT-GGU*, *trnL-UAG*--*ccsA* and one gene *ycf3*, which could be used as potential DNA barcodes for the genus. Future studies will focus on identifying adaptive codon sites in positively-selected genes and correlating those with the habitats and environmental conditions and validation of the proposed barcodes by including more *Limonium* species.

## Supplementary materials

Supplementary material 1 The output of the repeat analysis of four *Limonium* plastomes Data type molecular analysis https://binary.pensoft.net/file/527175This dataset is made available under the Open Database License (http://opendatacommons.org/licenses/odbl/1.0/). The Open Database License (ODbL) is a license agreement intended to allow users to freely share, modify, and use this Dataset while maintaining this same freedom for others, provided that the original source and author(s) are credited.Ashwini M. Darshetkar, Satish Maurya, Changyoung Lee, Badamtsetseg Bazarragchaa, Gantuya Batdelger, Agiimaa Janchiv, Eun Ju Jeong, Sangho Choi, Ritesh Kumar Choudhary, Soo-Yong Kim

Supplementary material 2 Relative Synonymous Codon Usage Data type molecular data https://binary.pensoft.net/file/527176This dataset is made available under the Open Database License (http://opendatacommons.org/licenses/odbl/1.0/). The Open Database License (ODbL) is a license agreement intended to allow users to freely share, modify, and use this Dataset while maintaining this same freedom for others, provided that the original source and author(s) are credited.Ashwini M. Darshetkar, Satish Maurya, Changyoung Lee, Badamtsetseg Bazarragchaa, Gantuya Batdelger, Agiimaa Janchiv, Eun Ju Jeong, Sangho Choi, Ritesh Kumar Choudhary, Soo-Yong Kim

Supplementary material 3 List of Positively selected genes Data type molecular data https://binary.pensoft.net/file/527177This dataset is made available under the Open Database License (http://opendatacommons.org/licenses/odbl/1.0/). The Open Database License (ODbL) is a license agreement intended to allow users to freely share, modify, and use this Dataset while maintaining this same freedom for others, provided that the original source and author(s) are credited.Ashwini M. Darshetkar, Satish Maurya, Changyoung Lee, Badamtsetseg Bazarragchaa, Gantuya Batdelger, Agiimaa Janchiv, Eun Ju Jeong, Sangho Choi, Ritesh Kumar Choudhary, Soo-Yong Kim

## References

[B1] Akhani H Malekmohammadi M Mahdavi P Gharibiyan A Chase MW (2013) Phylogenetics of the Irano-Turanian taxa of *Limonium* (Plumbaginaceae) based on ITS nrDNA sequences and leaf anatomy provides evidence for species delimitation and relationships of lineages. Botanical Journal of the Linnean Society 171(3): 519–550. 10.1111/boj.12015

[B2] Amiryousefi A Hyvönen J Poczai P (2018) IRscope: An online program to visualize the junction sites of chloroplast genomes. Bioinformatics 34(17): 3030–3031. 10.1093/bioinformatics/bty220 29659705

[B3] Andrews S (2010) Babraham bioinformatics-FastQC a quality control tool for high throughput sequence data. https://www.bioinformatics.babraham.ac.uk/projects/fastqc/

[B4] Bae M-J Karadeniz F Lee S-G Seo Y Kong C-S (2016) Inhibition of MMP-2 and MMP-9 Activities by *Limonium tetragonum* Extract. Preventive Nutrition and Food Science 21(1): 38–43. 10.3746/pnf.2016.21.1.38 27069904 PMC4827633

[B5] Beier S Thiel T Münch T Scholz U Mascher M (2017) MISA-web: A web server for microsatellite prediction. Bioinformatics 33(16): 2583–2585. 10.1093/bioinformatics/btx198 28398459 PMC5870701

[B6] Boissier E (1848) Plumbaginales. In: de Candolle AP (Ed.) Prodromussystematis naturalis regni vegetabilis, Treuttel et Wurz, Paris, France 12: 617–696.

[B7] Campagna ML Downie SR (1998) The Intron in Chloroplast Gene rpl16 is missing from the flowering plant families Geraniaceae, Goodeniaceae, and Plumbaginaceae. Transactions of the Illinois State Academy of Science. Illinois State Academy of Science 91: 1–11.

[B8] Chase MW Christenhusz MJM Fay MF Byng JW Judd WS Soltis DE Mabberley DJ Sennikov AN Soltis PS Stevens PF (2016) An update of the Angiosperm Phylogeny Group classification for the orders and families of flowering plants: APG IV. Botanical Journal of the Linnean Society 181(1): 1–20. 10.1111/boj.12385

[B9] Cowan RMJ Ingrouille M Lledó MD (1998) The taxonomic treatment of agamosperms in the genus *Limonium* Mill. (Plumbaginaceae). Folia Geobotanica 33(3): 353–366. 10.1007/BF03216212

[B10] Daniell H Lin C-S Yu M Chang W-J (2016) Chloroplast genomes: Diversity, evolution, and applications in genetic engineering. Genome Biology 17(1): e134. 10.1186/s13059-016-1004-2 PMC4918201 27339192

[B11] Darshetkar AM Datar MN Tamhankar S Li P Choudhary RK (2019) Understanding evolution in Poales: Insights from Eriocaulaceae plastome. PLoS ONE 14(8): e0221423. 10.1371/journal.pone.0221423 PMC6701780 31430346

[B12] Dierckxsens N Mardulyn P Smits G (2017) NOVOPlasty: De novo assembly of organelle genomes from whole genome data. Nucleic Acids Research 45: e18–e18. 10.1093/nar/gkw955 PMC5389512 28204566

[B13] Gao F Chen C Arab DA Du Z He Y Ho SYW (2019) EasyCodeML: A visual tool for analysis of selection using CodeML. Ecology and Evolution 9(7): 3891–3898. 10.1002/ece3.5015 31015974 PMC6467853

[B14] Gompert Z Mock KE (2017) Detection of individual ploidy levels with genotyping-by-sequencing (GBS) analysis. Molecular Ecology Resources 17(6): 1156–1167. 10.1111/1755-0998.12657 28150424

[B15] Hernández-Ledesma P Berendsohn WG Borsch T Von Mering S Akhani H Arias S Castañeda-Noa I Eggli U Eriksson R Flores-Olvera H (2015) A taxonomic backbone for the global synthesis of species diversity in the angiosperm order Caryophyllales. Willdenowia 45(3): 281–383. 10.3372/wi.45.45301

[B16] Jansen RK Saski C Lee S-B Hansen AK Daniell H (2011) Complete Plastid Genome Sequences of Three Rosids (*Castanea*, *Prunus*, *Theobroma*): Evidence for at least two independent transfers of rpl22 to the nucleus. Molecular Biology and Evolution 28(1): 835–847. 10.1093/molbev/msq261 20935065 PMC3108605

[B17] Jiang P Shi F-X Li M-R Liu B Wen J Xiao H-X Li L-F (2018) Positive selection driving cytoplasmic genome evolution of the medicinally important Ginseng plant genus *Panax*. Frontiers of Plant Science 9: e359. 10.3389/fpls.2018.00359 PMC5893753 29670636

[B18] Katoh K Standley DM (2013) MAFFT Multiple Sequence Alignment Software Version 7: Improvements in Performance and Usability. Molecular Biology and Evolution 30(4): 772–780. 10.1093/molbev/mst010 23329690 PMC3603318

[B19] Kong C-S Um Y-R Lee J-I Kim Y-A Lee J-S Seo Y-W (2008) Inhibition effects of extracts and its solvent fractions isolated from *Limonium tetragonum* on growth of human cancer cells. KSBB Journal 23: 177–182.

[B20] Koutroumpa K Theodoridis S Warren BH Jiménez A Celep F Doğan M Romeiras MM Santos‐Guerra A Fernández‐Palacios JM Caujapé‐Castells J Moura M de Sequeira MM Conti E (2018) An expanded molecular phylogeny of Plumbaginaceae, with emphasis on *Limonium* (sea lavenders): Taxonomic implications and biogeographic considerations. Ecology and Evolution 8(24): 12397–12424. 10.1002/ece3.4553 30619554 PMC6308857

[B21] Kubitzki K (1993) The Families and Genera of Vascular Plants: Flowering Plants: Dicotyledons, Magnoliid, Hamamelid and Caryophyllid Families. Springer, Berlin, Heidelberg & New York, 653 pp.

[B22] Kurtz S Choudhuri JV Ohlebusch E Schleiermacher C Stoye J Giegerich R (2001) REPuter: The manifold applications of repeat analysis on a genomic scale. Nucleic Acids Research 29(22): 4633–4642. 10.1093/nar/29.22.4633 11713313 PMC92531

[B23] Li J Liu H Mao S (2016) Adaptive evolution of ndhF gene in the genus *Rheum* (Polygonaceae). Guihaia 36: 101–106.

[B24] Li J Zhao C Zhang M Yuan F Chen M (2019) Exogenous melatonin improves seed germination in *Limonium bicolor* under salt stress. Plant Signaling & Behavior 14(11): 1659705. 10.1080/15592324.2019.1659705 31460852 PMC6804724

[B25] Li J Xu B Yang Q Wang T Zhu Q Lin Y Liu Z-L (2020) The complete chloroplast genome sequence of *Limonium sinense* (Plumbaginaceae). Mitochondrial DNA. Part B, Resources 5(1): 556–557. 10.1080/23802359.2019.1710286 PMC7748627 33366644

[B26] Lledó MD Crespo MB Cameron KM Fay MF Chase MW (1998) Systematics of Plumbaginaceae based upon cladistic analysis of *rbcL* sequence data. Systematic Botany 23(1): 21–29. 10.2307/2419571

[B27] Lledó MD Karis PO Crespo MB Cameron KM Fay MF Chase MW (2001) Phylogenetic position and taxonomic status of the genus *Aegialitis* and subfamilies Staticoideae and Plumbaginoideae (Plumbaginaceae): Evidence from plastid DNA sequences and morphology. Plant Systematics and Evolution 229(1–2): 107–124. 10.1007/s006060170021

[B28] Lledó MD Crespo MB Fay MF Chase MW (2005) Molecular phylogenetics of *Limonium* and related genera (Plumbaginaceae): Biogeographical and systematic implications. American Journal of Botany 92(7): 1189–1198. 10.3732/ajb.92.7.1189 21646141

[B29] Lledó MD Karis PO Crespo MB Fay MF Chase MW (2011) Endemism and evolution in Macaronesian and Mediterranean *Limonium*. In: Bramwell D Caujapé-Castells J (Eds) The Biology of Island Floras.Cambridge University Press, Cambridge, 325–337. 10.1017/CBO9780511844270.014

[B30] Logacheva MD Samigullin TH Dhingra A Penin AA (2008) Comparative chloroplast genomics and phylogenetics of Fagopyrum esculentum ssp. ancestrale – A wild ancestor of cultivated buckwheat. BMC Plant Biology 8(1): e59. 10.1186/1471-2229-8-59 PMC2430205 18492277

[B31] Lohse M Drechsel O Kahlau S Bock R (2013) OrganellarGenomeDRAW – A suite of tools for generating physical maps of plastid and mitochondrial genomes and visualizing expression data sets. Nucleic Acids Research 41(W1): W575–W581. 10.1093/nar/gkt289 PMC3692101 23609545

[B32] Lowe TM Chan PP (2016) tRNAscan-SE On-line: Integrating search and context for analysis of transfer RNA genes. Nucleic Acids Research 44(W1): W54–W57. 10.1093/nar/gkw413 PMC4987944 27174935

[B33] Lu C Feng Z Yuan F Han G Guo J Chen M Wang B (2020) The SNARE protein LbSYP61 participates in salt secretion in *Limonium bicolor*. Environmental and Experimental Botany 176: e104076. 10.1016/j.envexpbot.2020.104076

[B34] Malekmohammadi M Akhani H Borsch T (2017) Phylogenetic relationships of *Limonium* (Plumbaginaceae) inferred from multiple chloroplast and nuclear loci. Taxon 66(5): 1128–1146. 10.12705/665.8

[B35] Maurya S Darshetkar AM Datar MN Tamhankar S Li P Choudhary RK (2020) Plastome data provide insights into intra and interspecific diversity and ndh gene loss in *Capparis* (Capparaceae). Phytotaxa 432(2): 206–220. 10.11646/phytotaxa.432.2.10

[B36] McKain MR Johnson MG Uribe‐Convers S Eaton D Yang Y (2018) Practical considerations for plant phylogenomics. Applications in Plant Sciences 6(3): e1038. 10.1002/aps3.1038 PMC5895195 29732268

[B37] Millen RS Olmstead RG Adams KL Palmer JD Lao NT Heggie L Kavanagh TA Hibberd JM Gray JC Morden CW Calie PJ Jermiin LS Wolfe KH (2001) Many parallel losses of infA from chloroplast DNA during angiosperm evolution with multiple independent transfers to the nucleus. The PLANT Cell 13(3): 645–658. 10.1105/tpc.13.3.645 11251102 PMC135507

[B38] Nguyen L-T Schmidt HA von Haeseler A Minh BQ (2015) IQ-TREE: A fast and effective stochastic algorithm for estimating Maximum-Likelihood phylogenies. Molecular Biology and Evolution 32(1): 268–274. 10.1093/molbev/msu300 25371430 PMC4271533

[B39] Ohwi J (1965) Flora of Japan (rev. ed.). Shibundo Co. Ltd., Tokyo, 344 pp.

[B40] Palacios C Rosselló JA González-Candelas F (2000) Study of the evolutionary relationships among *Limonium* Species (Plumbaginaceae) using nuclear and cytoplasmic molecular markers. Molecular Phylogenetics and Evolution 14(2): 232–249. 10.1006/mpev.1999.0690 10679157

[B41] POWO (2021) Plants of the World Online. Facilitated by the Royal Botanic Gardens, Kew. http://www.plantsoftheworldonline.org/ [accessed 15 February 2021]

[B42] Price MN Dehal PS Arkin AP (2010) FastTree 2 – Approximately Maximum-Likelihood Trees for Large Alignments. PLoS ONE 5(3): e9490. 10.1371/journal.pone.0009490 PMC2835736 20224823

[B43] Róis AS Sádio F Paulo OS Teixeira G Paes AP Espírito-Santo D Sharbel TF Caperta AD (2016) Phylogeography and modes of reproduction in diploid and tetraploid halophytes of *Limonium* species (Plumbaginaceae): Evidence for a pattern of geographical parthenogenesis. Annals of Botany 117(1): 37–50. 10.1093/aob/mcv138 26424783 PMC4701142

[B44] Rozas J Ferrer-Mata A Sánchez-DelBarrio JC Guirao-Rico S Librado P Ramos-Onsins SE Sánchez-Gracia A (2017) DnaSP 6: DNA sequence polymorphism analysis of large data sets. Molecular Biology and Evolution 34(12): 3299–3302. 10.1093/molbev/msx248 29029172

[B45] Schwartz S Elnitski L Li M Weirauch M Riemer C Smit A Program NCS Green ED Hardison RC Miller W (2003) MultiPipMaker and supporting tools: Alignments and analysis of multiple genomic DNA sequences. Nucleic Acids Research 31(13): 3518–3524. 10.1093/nar/gkg579 12824357 PMC168985

[B46] Thomas GWC Ather SH Hahn MW (2017) Gene-Tree Reconciliation with MUL-Trees to resolve polyploidy events. Systematic Biology 66(6): 1007–1018. 10.1093/sysbio/syx044 28419377

[B47] Tillich M Lehwark P Pellizzer T Ulbricht-Jones ES Fischer A Bock R Greiner S (2017) GeSeq – versatile and accurate annotation of organelle genomes. Nucleic Acids Research 45(W1): W6–W11. 10.1093/nar/gkx391 PMC5570176 28486635

[B48] Tomoko O (1995) Synonymous and nonsynonymous substitutions in mammalian genes and the nearly neutral theory. Journal of Molecular Evolution 40(1): 56–63. 10.1007/BF00166595 7714912

[B49] Walker JF Yang Y Feng T Timoneda A Mikenas J Hutchison V Edwards C Wang N Ahluwalia S Olivieri J Walker‐Hale N Majure LC Puente R Kadereit G Lauterbach M Eggli U Flores‐Olvera H Ochoterena H Brockington SF Moore MJ Smith SA (2018) From cacti to carnivores: Improved phylotranscriptomic sampling and hierarchical homology inference provide further insight into the evolution of Caryophyllales. American Journal of Botany 105(3): 446–462. 10.1002/ajb2.1069 29738076

[B50] Wu Z Raven PH (1996) Flora of China. Science Press (Beijing) & Missouri Botanical Garden Press (St. Louis), 15: 1–387.

[B51] Yang Z (1998) Likelihood ratio tests for detecting positive selection and application to primate lysozyme evolution. Molecular Biology and Evolution 15(5): 568–573. 10.1093/oxfordjournals.molbev.a025957 9580986

[B52] Yao G Jin J-J Li H-T Yang J-B Mandala VS Croley M Mostow R Douglas NA Chase MW Christenhusz MJM Soltis DE Soltis PS Smith SA Brockington SF Moore MJ Yi T-S Li D-Z (2019) Plastid phylogenomic insights into the evolution of Caryophyllales. Molecular Phylogenetics and Evolution 134: 74–86. 10.1016/j.ympev.2018.12.023 30735725

[B53] Zhang X Xu Y Liu X (2020) Complete plastome sequence of *Limonium aureum*, a medicinal and ornamental species in China. Mitochondrial DNA. Part B, Resources 5(1): 333–334. 10.1080/23802359.2019.1703608 PMC7748777 33366544

[B54] Zhong Q Yang S Sun X Wang L Li Y (2019) The complete chloroplast genome of the Jerusalem artichoke (*Helianthus tuberosus* L.) and an adaptive evolutionary analysis of the ycf2 gene. PeerJ 7: e7596. 10.7717/peerj.7596 PMC6718157 31531272

[B55] Zhu A Guo W Gupta S Fan W Mower JP (2016) Evolutionary dynamics of the plastid inverted repeat: The effects of expansion, contraction, and loss on substitution rates. The New Phytologist 209(4): 1747–1756. 10.1111/nph.13743 26574731

